# Quantification and localization of oncogenic receptor tyrosine kinase variant transcripts using molecular inversion probes

**DOI:** 10.1038/s41598-018-25328-5

**Published:** 2018-05-04

**Authors:** Corina N. A. M. van den Heuvel, Arvid I. Das, Tessa de Bitter, Femke Simmer, Thomas Wurdinger, Miguel Angel Molina-Vila, William P. J. Leenders

**Affiliations:** 1 Department of Biochemistry, Radboud Institute for Molecular Life Sciences, University Medical Centre, Nijmegen, 6525 GA The Netherlands; 2 Department of Pathology, Radboud University Medical Centre, Nijmegen, 6500 HB The Netherlands; 3 VU Medical Centre Amsterdam, Department of Neurosurgery, Amsterdam, The Netherlands; 4 PangaeaOncology, Laboratory of Oncology, University Hospital Quiron Dexeus, Barcelona, Spain

## Abstract

Oncogenic membrane receptor tyrosine kinases such as MET and EGFR, or auto-active variants thereof, are important targets for cancer precision therapy. Targeted inhibition of these oncogenic receptors however invariably leads to resistance, resulting from acquisition of resistance-inducing mutations or from selective outgrowth of a priori resistant tumour cells. Most applied molecular protocols cannot distinguish between intracellular and intercellular heterogeneity of oncogene (variant) expression, which may lead to misinterpretation of the molecular make-up of a cancer and suboptimal application of targeted therapies. We here combined two related techniques to allow semiquantitative and localized *in situ* detection of specific transcript splice variants using single molecule molecular inversion probe (smMIP)-based next generation sequencing and padlock probe-based rolling circle amplification, respectively. We show highly specific padlock probe-based multiplex detection of MET, MET^Δ7-8^ and MET^Δ14^ transcripts, lacking exons 7–8 and exon 14 respectively, and of EGFR and the auto-active EGFRvIII, lacking exons 2–7. The combination of quantitative transcript variant detection with smMIPs and transcript localization using padlock probes can be used for detection of oncogenic transcripts on the single-cell level, allowing study of tumour heterogeneity. Visualization of tumour heterogeneity can shed light on the biology underlying drug resistance and potentially improve targeted therapeutics.

## Introduction

Membrane receptor tyrosine kinases, e.g. EGFR, FGFR, IGF1-R, MET, AXL, KIT, RON, VEGFRs and PDGFRs^1^, have become important targets for cancer precision therapy. Although inhibition of the oncogenic products of these genes frequently leads to initial responses, experience is that eventually treatment-resistant tumours develop. Treatment failure may result from intrinsic intratumoural heterogeneity or from acquisition of resistance-inducing mutations^2–4^. An example of the latter is acquisition of EGFR^T790M^ or EGFR^C797S^ mutations in non small cell lung cancers upon treatment with the EGFR tyrosine kinase inhibitor erlotinib^5,6^. Novel precision medicines have been developed that specifically inhibit these mutant EGFR variants^7^. Formally however, it is unclear whether such aberrations are acquired de novo or whether a priori resistant cells pre-existed in the primary tumour, experiencing a growth advantage and eventually replacing the treatment-sensitive tumour bulk. This is a highly important question, since in the latter case patients should be treated with combination therapy comprising relevant tyrosine kinase inhibitors at start. Therefore it is important to investigate heterogeneity of expression of tyrosine kinases, but also other oncogenes, in tumours in more detail.

Molecular research as well as diagnostics of cancer currently consists of analysis of genomic DNA which is extracted from cancer tissues that also contain non-tumour cells. In these situations it is impossible to assign wild-type alleles to cancer- or stromal cells, or to detect tumour heterogeneity. Examples of oncogenic receptors are the MET and EGFR oncogenes, of which different auto-active variants exist. The MET^Δ7-8^ variant results from a ~2 Kb intragenic deletion which results in spliced mRNA lacking exons 7 and 8. This variant, that misses only 80 amino acids in the ectodomain, is expressed in 6% of high-grade gliomas, remains intracellularly and its activation is ligand independent^8^. MET^Δ14^ is a splice variant lacking exon 14, a result of exon-skipping point mutations in splice sites^9,10^, and is hyperactive due to increased protein stability. MET^Δ14^ is detected in 0.4% of glioma and 3–4% of non-small cell lung carcinoma (NSCLC) cases^9–12^. Increased MET (variant) expression has been described for NSCLC under gefitinib treatment and may represent a rescue kinase for EGFR inhibition^13–16^.

A second example of oncogene aberration is EGFRvIII. Whereas EGFR is amplified in 50% of glioblastomas, half of these events is accompanied by an intragenic deletion, resulting in EGFR transcripts lacking exons 2–7^9,17^. Protein products of this transcript lack a large part of the extracellular domain and are auto-active. Because the EGFRvIII protein contains a neo-epitope at the exon 1–8 junction, it is tumour specific and therefore of considerable interest for targeted therapies^18,19^. In glioblastoma, expression of this variant emerges after initial EGFR amplification and is heterogeneous, being more prominent in diffuse infiltrating areas of the brain^17^. Importantly, this spatial heterogeneity cannot be inferred from genetic analysis. This highlights the need for expression analysis on the single-cell level.

**Figure 1:**
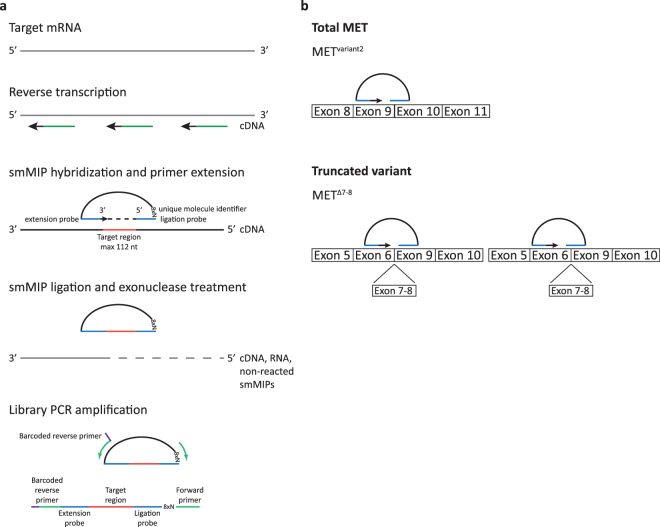
Quantitative detection of transcript splice variants using smMIPs. (**a**) Schematic overview of the method. mRNA is reverse transcribed into cDNA using random primers. SmMIP probes hybridize to the target region of interest, leaving a gap of ~112 nucleotides. The gap is filled by primer extension and ligation, whereafter all remaining linear smMIPs, RNA and cDNA are removed by exonuclease treatment. All circular smMIPs are PCR amplified using a unique barcoded reverse primer for each sample. Resulting reads are mapped against reference transcriptomes, normalized to the total read count within a sample (FPM), and averaged per transcript variant. (**b**) Design of smMIP probes. To determine the total expression of a gene, smMIPs were designed against shared parts of the transcript of interest (upper panel). For specific detection of splice variants smMIPs were designed to target variant-specific exon-exon junctions (for example the exon6–9 junction for MET^Δ7-8^), either covering the junction with the gap or the ligation/extension probe (lower panel left and right, respectively). Note that for graphical representation, the figure is not to scale.

**Figure 2:**
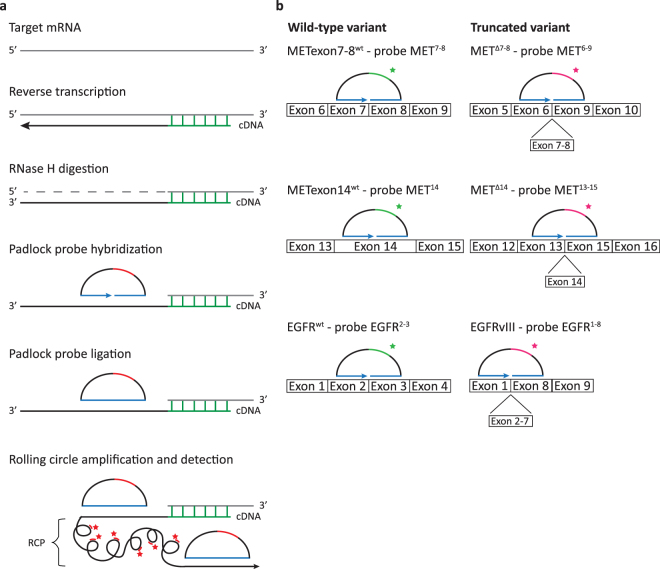
*In situ* detection of whole-exon deletion splice variants using padlock probes. (**a**) Schematic overview of the method. mRNA is converted into localized cDNA molecules, which can be targeted through padlock probe hybridization and rolling circle amplification. Rolling circle products (RCPs) can then be detected with fluorescently labeled probes. Nucleic acid bridges in the BNA primer are depicted in green, padlock probe target sites are depicted in blue and padlock probe detection sites are depicted in red. (**b**) Design of padlock probes targeting METexon7–8^wt^, MET^Δ7-8^, METexon14^wt^, MET^Δ14^, EGFR^wt^ and EGFRvIII. For specific detection of transcript splice variants, padlock probes were designed to target the mutation-specific exon-exon junction (detected with magenta fluorescence). For distinctive detection of their associated wild-type variants, padlock probes were targeted at the exon(s) missing in the exon deletion transcripts (detected with green fluorescence). Note that for graphical representation, the figure is not to scale.

In this study, we combine single molecule molecular inversion probe (smMIP) targeted transcriptome sequencing^20,21^ (Fig. 1) with *in situ* detection of transcript splice variants using padlock probe-based rolling circle amplification^22–26^ (Fig. 2). We show that we are able to specifically and semiquantitatively detect transcript variants in RNA samples from both cell lines and xenografts using smMIPs, and we demonstrate how *in situ* padlock probe rolling circle amplification adds essential information on transcript localization and tumour heterogeneity.

## Results

**Figure 3:**
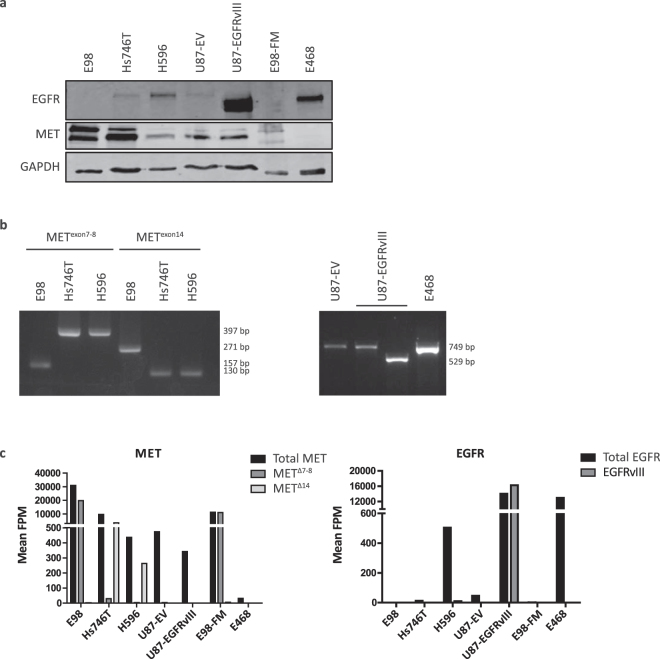
Expression levels and mutation status of MET and EGFR in relevant cell lines and xenografts. (**a**) western blot of cell lines E98, Hs746T and H596, U87-EV, U87-EGFRvIII, and xenografts E98-FM and E468, showing relative (total) MET and EGFR expression. Note that MET shows two proteins of different sizes, corresponding to the preform and the processed form of the protein^8^. (**b**) Left: MET PCR on cell lines E98, Hs746T and H596 to confirm MET^Δ7-8^ and MET^Δ14^ mutation status. Right: EGFR PCR on cell lines U87-EV and U87-EGFRvIII, and xenograft E468, to confirm EGFR^wt^ and EGFRvIII status. (**c**) SmMIP targeted transcriptome profiling of MET (left) and EGFR (right) quantifies variant-specific expression levels in cell lines and xenografts. While E98 cells and xenografts express the MET^Δ7-8^ variant, Hs746T and H596 predominantly express the MET^Δ14^ variant. U87 cells express MET^wt^ transcripts. EGFR^wt^ transcript expression is present in Hs746T, H596, U87 and E468 cells and xenografts, while the EGFRvIII variant is only present in the U87-EGFRvIII cell line.

To investigate whether padlock assays can be used for specific *in situ* detection of oncogene transcripts, we here examined expression of MET and EGFR and auto-active splice variants of these tyrosine kinases, in a cohort of cell lines and xenografts with defined EGFR and MET expression. To set up the technique we used cell lines E98 and its corresponding xenograft (E98-FM, MET^Δ7-8^ amplification)^8^, cell lines Hs746T (MET^Δ14^, high expression), H596 (MET^Δ14^, low expression), U87-EV (EGFR^wt^, low expression), U87-EGFRvIII (over-expression)^27^, and xenograft E468 (EGFR amplification)^28^. Relative protein expression levels of MET and EGFR in the different cell lines and xenografts are shown in Fig. 3a. By PCR analysis we confirmed the MET transcript status of E98, Hs746T and H596, and EGFR transcript mutation status of E468, U87-EV and U87-EGFRvIII (Fig. 3b). We semiquantitatively determined transcript variant-specific expression levels of MET and EGFR in all cell lines and xenografts using smMIPs (Fig. 3c).

**Figure 4:**
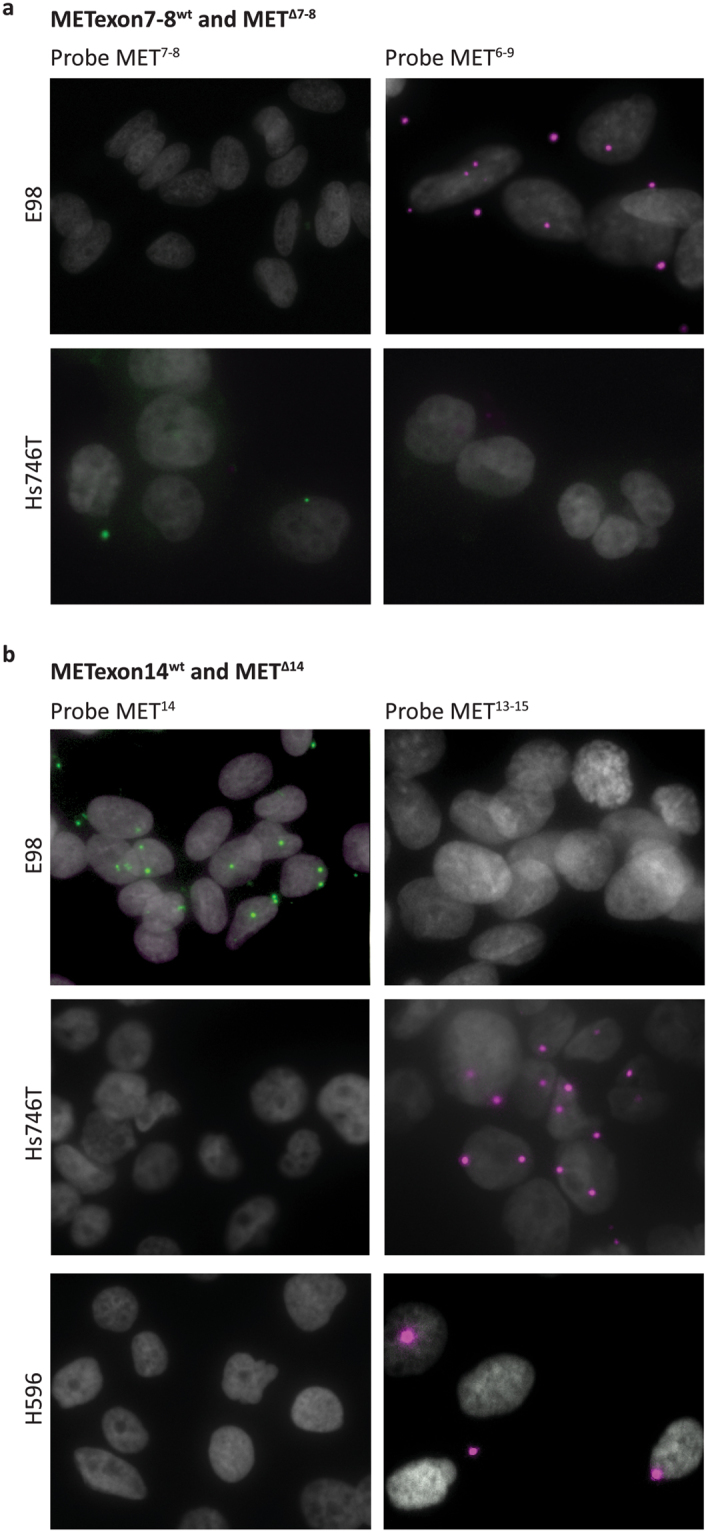
Single-probe *in situ* mutation detection of MET using padlock probe rolling circle amplification on cell lines. (**a**) *In situ* detection of METexon7–8^wt^ and MET^Δ7-8^ transcripts in E98 and Hs746T cells using padlock probes MET^7,8^ and MET^6–9^, respectively. METexon7–8^wt^ transcripts are displayed as green RCPs, MET^Δ7-8^ transcripts are displayed as magenta RCPs. Cell nuclei are shown in grey. Original magnification 40x. (**b**) *In situ* detection of METexon14^wt^ and MET^Δ14^ transcripts in E98, Hs746T and H596 cell lines using padlock probes MET^14^ and MET^13–15^, respectively. METexon14^wt^ transcripts are displayed as green RCPs, MET^Δ14^ transcripts are displayed as magenta RCPs. Cell nuclei are shown in grey. Original magnification 40x.

We first tested the specificity of the MET-targeting padlock probes on cell lines with known MET status. Single-probe assays with padlock probe MET^7,8^ resulted in fluorescent signal in Hs746T, but not in E98 cells. Conversely, single-probe assays with padlock probe MET^6–9^ resulted in fluorescent signal in E98, but not in Hs746T cells (Fig. 4a). These results are in line with the fact that E98 cells exclusively express MET^Δ7-8^, while Hs746T cells only express METexon7–8^wt^ transcripts, confirming the specificity of MET^7,8^ and MET^6–9^ probes. Similarly, we performed single-probe padlock assays with the MET^14^ and MET^13–15^ probes on E98 cells in which no MET^Δ14^ is expressed, and Hs746T and H596 cell lines, expressing MET^Δ14^. As expected, in E98 cells METexon14^wt^ transcripts were readily detected with probe MET^14^, while the assay was negative when performed with the MET^13–15^ probe (Fig. 4b upper panel). Conversely, Hs746T and H596 cells were positive when assayed with the padlock MET^13–15^ probe, and negative with the MET^14^ probe (Fig. 4b middle and lower panels). Numbers of fluorescent spots in H596 cells were lower than in Hs746T cells, in agreement with the lower levels of MET protein that we detected by western blot in these cell lines (Fig. 3a) and lower levels of MET transcript as detected by smMIP-sequencing (Fig. 3c).

**Figure 5:**
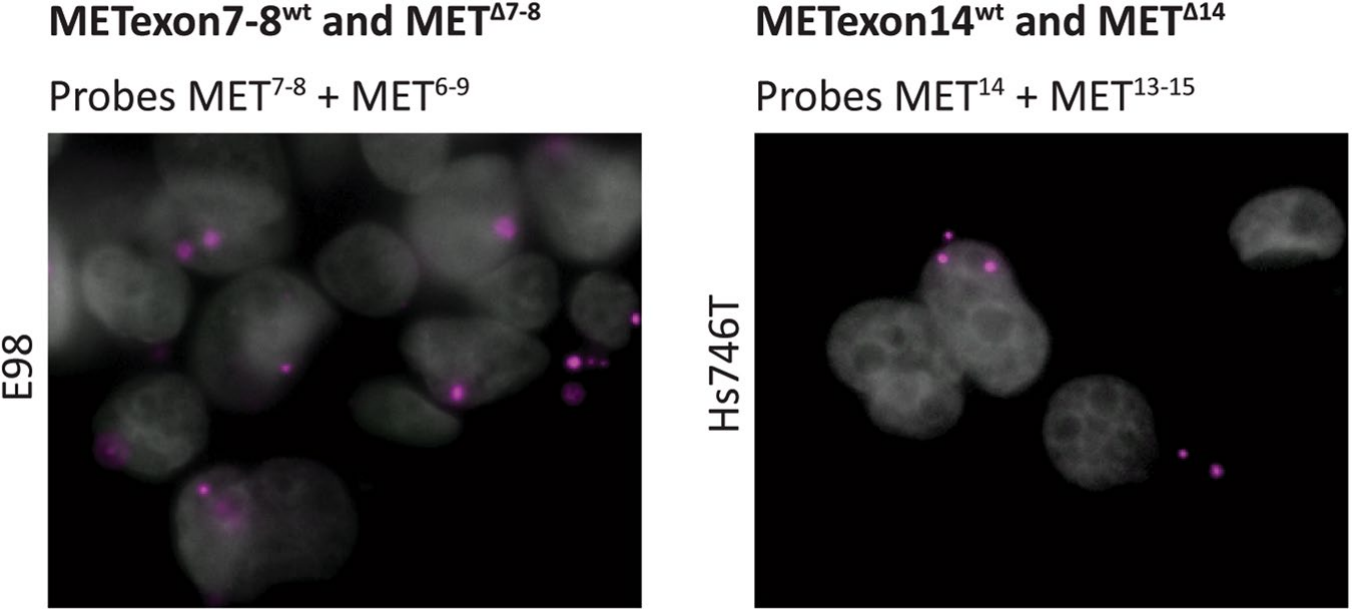
Multi-plex *in situ* mutation detection of MET using padlock probe rolling circle amplification on cell lines. *In situ* duo-probe detection of METexon7–8^wt^ and MET^Δ7-8^ transcripts in E98 cells (left) and METexon14^wt^ and MET^Δ14^ transcripts in Hs746T cells (right) using padlock probes MET^7,8^ and MET^6–9^, and MET^14^ and MET^13–15^, respectively. METexon7–8^wt^ and METexon14^wt^ transcripts are displayed as green RCPs, MET^Δ7-8^ and MET^Δ14^ transcripts are displayed as magenta RCPs. Cell nuclei are shown in grey. Original magnification 40x.

Having confirmed the specificity of these probes, we subjected E98 and Hs746T cells to a combination of MET^7,8^ and MET^6–9^ probes, and MET^14^ and MET^13–15^ probes, respectively. This duo-probe padlock assay allows duplex detection of wild-type (detected in green) and mutant variants (detected in magenta) of the same RNA transcript simultaneously. The selectivity of the padlock probes was preserved when mutant-specific probes were applied together with their corresponding wild-type probe, and also the efficiency was not notably affected (Fig. 5).

**Figure 6:**
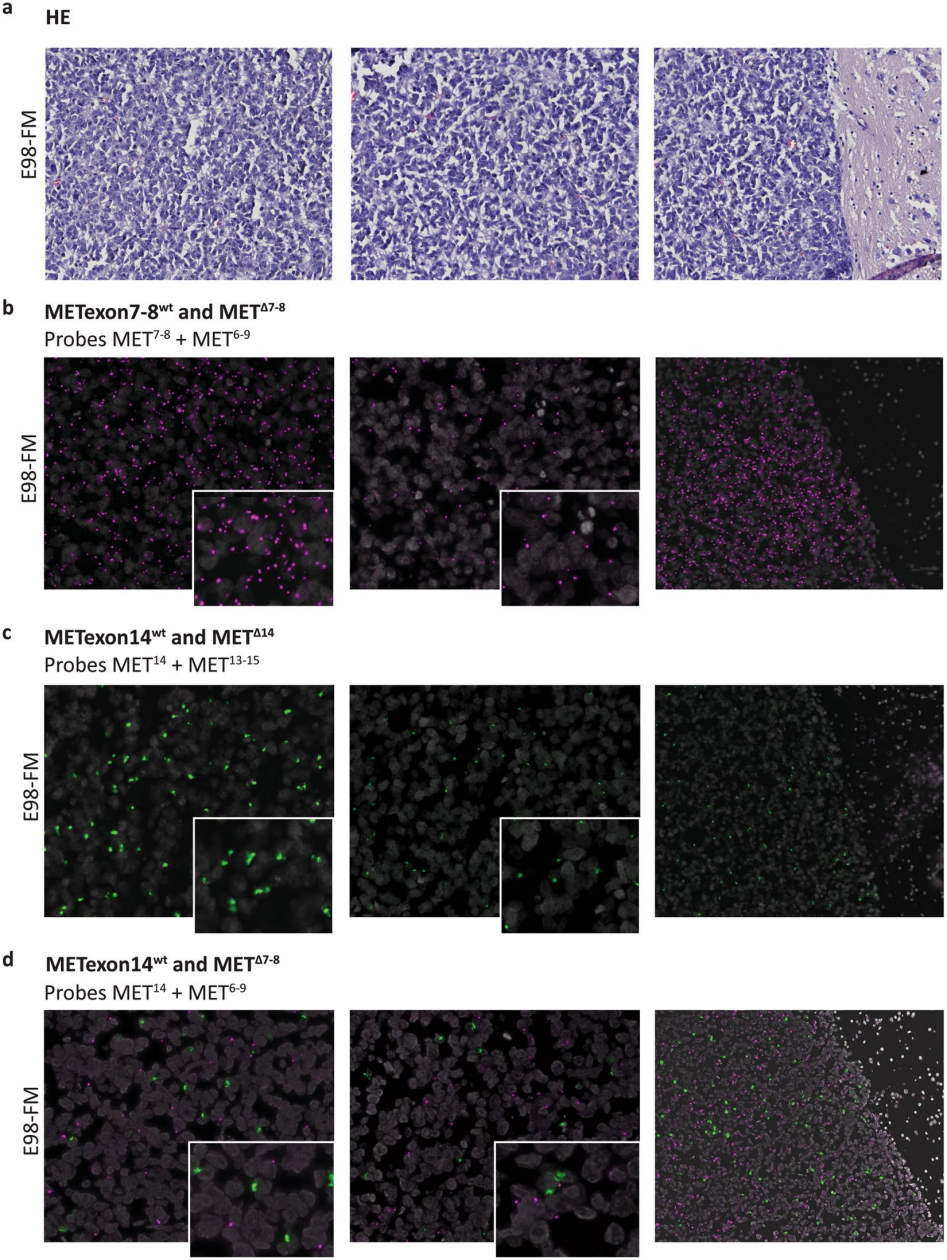
Multi-plex *in situ* mutation detection of MET using padlock probe rolling circle amplification on E98-FM FFPE xenograft tissue. (**a**) Serial H&E stainings. (**b**) *In situ* duo-probe detection of METexon7–8^wt^ and MET^Δ7-8^ transcripts using padlock probes MET^7,8^ and MET^6–9^, respectively. (**c**) *In situ* duo-probe detection of METexon14^wt^ and MET^Δ14^ transcripts using padlock probes MET^14^ and MET^13–15^, respectively. (**d**) *In situ* duo-probe detection of METexon14^wt^ and MET^Δ7-8^ transcripts using padlock probes MET^14^ and MET^6–9^, respectively. Wild-type transcripts are displayed as green RCPs, mutant transcripts are displayed as magenta RCPs. Cell nuclei are shown in grey. Note that in all assays the tumour border emphasizes the specificity of the padlock probes, showing no fluorescent detection spots in normal brain tissue (right figures, original magnification 20x). Compare the left and middle panels for each assay, visualizing differences in transcript expression in different tumour areas. Inserts give a zoomed view of the underlying image. Original magnification 40x.

After successful and specific detection of MET splice variants in cell lines, we tested our duo-probe padlock assay on FFPE xenograft material. We subjected serial E98-FM FFPE tissue slides, carrying the MET^Δ7-8^ mutation but with a wild type exon 14 allele, to duo-probe padlock assays using a combination of probes MET^6–9^ and MET^7,8^ (Fig. 6b), MET^13–15^ and MET^14^ (Fig. 6c), or MET^14^ and MET^6–9^ (Fig. 6d). H&E sections serial to the padlock assay tissues are depicted in Fig. 6a. Both MET^Δ7-8^ and METexon14^wt^ transcripts were specifically detected in E98 xenograft tissue, without detection spots for METexon7–8^wt^ or MET^Δ14^ transcripts, respectively (Fig. 6b,c). We could visualize different tumour areas with high and low expression of the targeted MET transcripts (Fig. 6b,c, left and middle panels, respectively). Note that normal tissue (right part of right panels) was completely negative. A double-positive padlock assay targeting METexon14^wt^ and MET^Δ7-8^ on E98 xenograft tissue showed specific detection of both transcripts simultaneously (Fig. 6d). Probe specificity is again highlighted at the tumour border (Fig. 6d, right panel). Although both padlock probes were readily identified in these sections, co-localization was only occasionally observed (see Supplementary Fig. 2, arrows). This is possibly due to the low sensitivity of the padlock probe assay. Of note, signals were slightly decreased in the double-positive assay as compared to the single-positive padlock assays. Although we could not quantify this, we attribute this to competition of padlock probes on the same transcript.

**Figure 7:**
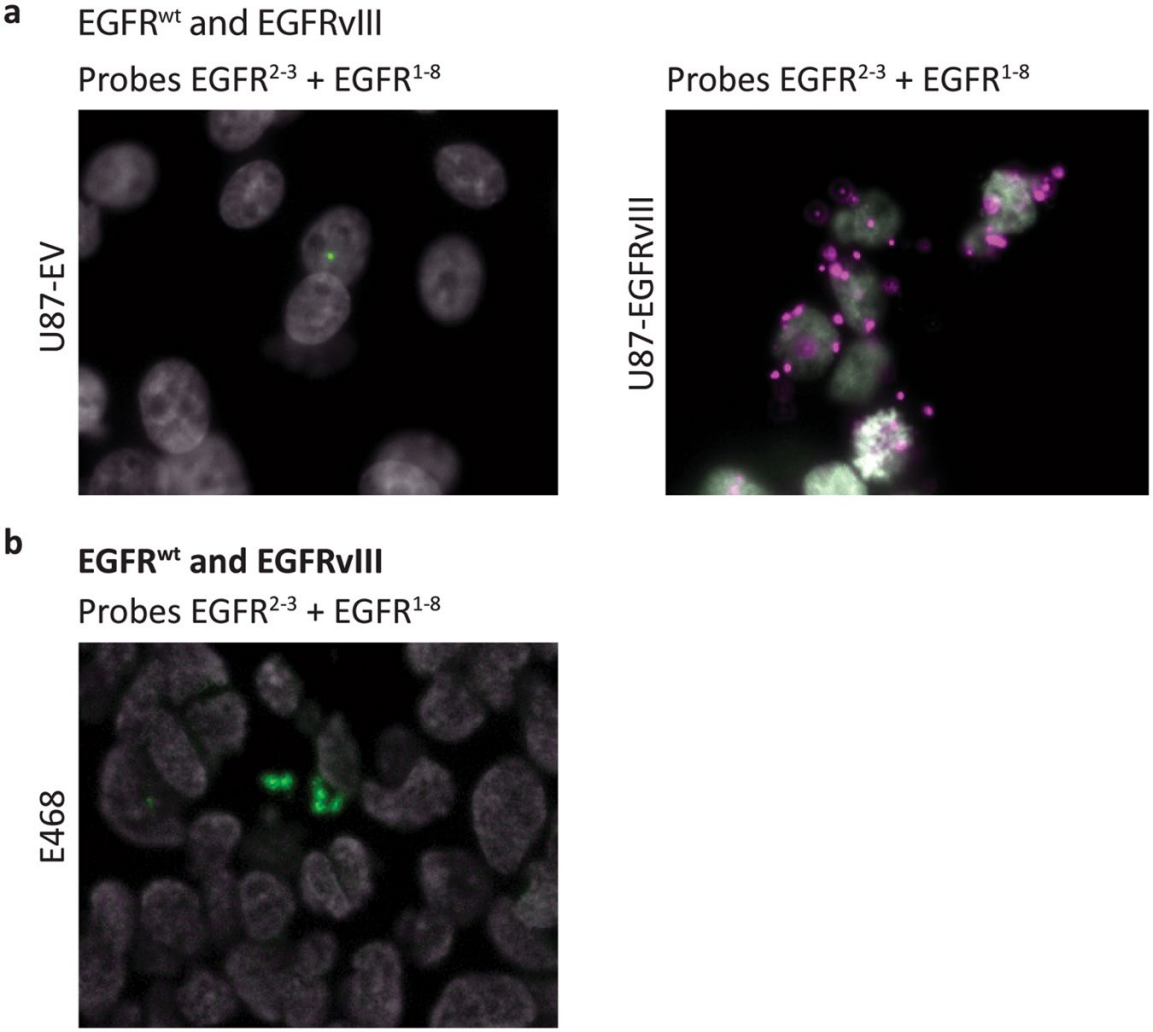
Multi-plex *in situ* mutation detection of EGFR using padlock probe rolling circle amplification on cell lines and E468 xenograft tissue. A) *In situ* duo-probe detection of EGFR^wt^ and EGFRvIII transcripts using padlock probes EGFR^2,3^ and EGFR^1–8^, on U87-EV and U87-EGFRvIII cells (original magnification 40x). B) *In situ* duo-probe detection of EGFR^wt^ and EGFRvIII transcripts using padlock probes EGFR^2,3^ and EGFR^1–8^, on E468 xenograft FFPE tissue (original magnification 20x). EGFR^wt^ transcripts are displayed as green RCPs, EGFRvIII transcripts are displayed as magenta RCPs. Cell nuclei are shown in grey.

Similar to the padlock assays targeting MET, we performed padlock assays targeting EGFR^wt^ and EGFRvIII transcripts. We subjected U87-EV (EGFR^wt^) and U87-EGFRvIII overexpressing cell lines to a duplex assay to detect EGFR^wt^ and EGFRvIII transcripts, using padlock probes EGFR^2,3^ (green) and EGFR^1–8^ (magenta), respectively. In U87-EV cells this assay resulted in fluorescent signal for EGFR^wt^, while EGFRvIII specific transcripts were abundantly detected in U87-EGFRvIII overexpressing cells (Fig. 7a). In U87-EGFRvIII cells no EGFR^wt^ signals were detected in the duplex assay, probably because of the low abundance of EGFR^wt^ transcripts in these cells, compared to high overexpression of EGFRvIII transcripts. Note that the number of EGFR^wt^ fluorescent spots in U87-EV cells is much lower than the number of EGFRvIII spots in U87-EGFRvIII cells, which corresponds to the difference in EGFR expression (Fig. 3a,c). We also subjected EGFR^wt^ expressing E468 xenograft tissue to a duplex EGFR padlock assay. Figure 7b shows specific detection of EGFR^wt^ transcripts.

## Discussion

To be able to investigate tumour heterogeneity with respect to oncogene expression, we applied padlock probe rolling circle amplification to indentify EGFR, MET and the whole-exon deletion splice variants of these oncogenes that play a role in, among others, glioma and NSCLC^1,5,16,29–31^. Simultaneous *in situ* detection of tyrosine kinase splice variants with their associated wild-type variants enables visualization of tumour heterogeneity in FFPE tissue sections, material which is routinely used in diagnostics and can be preserved for years.

In recent years, articles have reported on the use of padlock probes for *in situ* analysis of single-nucleotide transcript variants^22,24^ and fusion transcripts^23^. Here, we adapted these protocols to allow *in situ* identification of whole-exon deletion splice variants. Probe specificity was validated in single-probe assays on cell lines with known MET or EGFR status, and was preserved in duplex padlock assays. As for now, a drawback is low sensitivity of padlock probe assays, possibly explaining the lack of co-localization of MET^14^ and MET^6–9^ probes in Fig. 6d. The distinction between intercellular and intracellular tumoural heterogeneity, with different oncogene variants being present within the same cell or with subclones of cells carrying different oncogene variants, respectively^2,3,32^, can therefore not be made. This distinction is important since cells co-expressing different tyrosine kinases that initiate similar signalling pathways, will respond differently to drugs than cells expressing only the targeted kinase. Low sensitivity of padlock probe assays has been described before^22,24,25^. However, the reason is not clear and may be related to any of the complex steps in the procedure, including fixation method/RNA quality, RNA secondary structures that hamper effective *in situ* reverse transcription, and the presence of single nucleotide polymorphisms (SNPs) impeding hybridisation of the BNA probe and the padlock probe. Improvement of sensitivity of the method is necessary to detect co-expression of different transcripts in one cancer cell. However, the high specificity of the padlock assay will allow the investigation of intratumoural, intercellular heterogeneity of splice variant transcript expression. To allow successful padlock assays on clinical tissue, the essentiality of RNA integrity probably requires that tumour tissue is fixated shortly after surgery or biopsy in proper preservatives.

For smMIP-based targeted RNA sequencing we have observed a variance in efficacy between different smMIPs targeting the same transcript. We have reported that the average of five smMIPs targeting one transcript gives a reliable estimate of the expression level of a transcript^21^. Because padlock assays rely on the hybridisation of one individual probe for each transcript variant, these are not suitable for quantification of transcript (variant) expression levels. *In situ* rolling circle amplification using padlock probes does allow visualization of intercellular heterogeneity within tumour tissues. In contrast, smMIP-based targeted RNA sequencing allows quantitative and specific detection of splice variants, but does not enable visualization of tumour heterogeneity and localization of specific transcripts. The combination of both techniques possibly allows differentiation between intrinsic and acquired transcript expression (induced tumour heterogeneity), and between intrinsic and acquired treatment resistance development. This is an interesting aspect for future studies and may give more insight into tumour resistance biology. This potentially has implications for targeted therapy, because patients with pre-existent resistant tumour clones may benefit significantly from combination therapies at start.

In conclusion, we here show the high value of combining two molecular inversion probe techniques. Using smMIP targeted transcriptomics we can quantitatively measure transcript variant-specific gene expression. Additionally, padlock probe rolling circle amplification enables specific and local visualization of transcript variants *in situ*, however with low efficacy. The technique of smMIP-based RNA splice variant sequencing can be easily expanded with smMIP probes to detect other transcript variants, and can serve as an important tool for detection of molecular heterogeneity and investigation of the role of splice variants in therapy resistance.

## Materials and Methods

### Cell lines and xenografts

Cell lines Hs746T and H596 were obtained from ATCC (Manassas, VA). Cell lines E98^28^, Hs746T, H596, U87-EV and U87-EGFRvIII (obtained from dr. Web Cavenee, Ludwig Cancer Inst., USA^27^) were cultured at 37 °C in the presence of 5% CO_2_ in Dulbecco’s Modified Eagle’s Medium (DMEM) containing 4.5 g/L glucose and 4 mM L-glutamin (Lonza, Basel, Switserland), 10% fetal calf serum (FCS, Gibco, Waltham, MA, USA) and 40 µg/ml gentamycin (Centrafarm, Ettenleur, The Netherlands). The MET^Δ7-8^-expressing astrocytoma cell line E98 and the EGFR-expressing astrocytoma line E468, as well as the generation of orthotopic xenografts thereof, has been described before^8,28^.

### Western blot

Cells were lysed in 1x RIPA buffer (Cell Signaling Technology, CST, Danvers, MA) with 1 mM phenylmethylsulfonyl fluoride (PMSF), according to manufacturer’s instructions. Protein lysates were subjected to electrophoresis on 10% SDS-PAGE gels and electroblotted onto nitrocellulose membranes (Whatman Optitran BA-S85, GE Healthcare, Little Chalfont, UK). After blocking aspecific binding sites in blocking buffer (1:1 PBS/Odyssey blocking buffer [LI-COR Biosciences, Lincoln, NE, USA]), blots were incubated o/n at 4 °C with primary antibodies: rabbit-anti-MET (1:2500, CST, #8198), rabbit-anti-EGFR D38B1 (1:2000, CST, #4267) and mouse-anti-GAPDH (1:5000, Abcam, ab8245). Primary antibodies were detected with appropriate IRDye680- or IRDye800-conjugated secondary antibodies (Invitrogen Molecular Probes, Waltham, MA, USA) incubated 1 hr at RT shielded from light. Signals were visualized using the Odyssey imaging system (LI-COR Biosciences, Lincoln, NE, USA).

### PCR

RNA was isolated from cell lines and snap-frozen xenograft tissue using TRIzol reagent (Life Technologies, ThermoFisher Scientific, Waltham, MA, USA) and reverse transcribed with Superscript II (Invitrogen, ThermoFisher Scientific, Waltham, MA, USA) using random hexamer primers, according to the manufacturer’s instructions. RT-PCR was performed using Amplitaq Gold 360 mastermix (Applied Biosystems, Life Technologies), which for PCR on MET was supplemented with 1 mM MgCl_2_ and 20 µg/ml BSA (both New England Biolabs, Ipswich, MA, USA). To distinguish MET^Δ7-8^ transcripts from their wild-type variant (METexon7–8^wt^), primers MET1997Fw (5′-CTCCTTGGAAATGAGAGCTG-3′, forward, located in exon 6) and MET2393Rv (5′-AGATGCTTGTCTCTCGGTTG-3′, reverse, located in exon 9) were used. This PCR results in a 397-bp amplicon for METexon7–8^wt^ and a 157-bp product for MET^Δ7-8^. To distinguish MET^Δ14^ transcripts and their wild-type variant (METexon14^wt^) primers MET2982Fw (5′- CAGGATTGATTGCTGGTGTTGTCTC-3′, forward, located in exon 13) and MET3252Rv (5′- CGGCATGAACCGTTCTGAGATG-3′, reverse, located in exon 15) were used, resulting in a PCR product of 271 bp for METexon14^wt^ and 130 bp for MET^Δ14^. To detect EGFR^wt^ transcripts primers EGFR819Fw (5′-GATATCACCATGCGACCCTCCGGG-3′, forward, located in exon 5) and EGFR1567Rv (5′-CGACTGCAAGAGAAAACTGA-3′, reverse, located in exon 12) were used, resulting in a 749-bp product for EGFR^wt^. For detection of EGFRvIII transcripts in U87-EGFRvIII cells, primers EGFR258Fw (5′-GATATCACCATGCGACCCTCCGGG-3′, forward, located in exon 1) and EGFR1567Rv (5′-CGACTGCAAGAGAAAACTGA-3′, reverse, located in exon 12) were used, resulting in a 529-bp product for EGFRvIII. PCR conditions were: initial denaturation of 3 min at 95 °C, followed by 36 cycles of denaturation at 95 °C, 30 s; annealing at 60 °C, 30 s; elongation at 72 °C, 30 s, and a final elongation step of 5 min at 72 °C.

### smMIP targeted RNA sequencing

RNA from cell lines and snap-frozen xenograft tissue was isolated and converted to cDNA as described under ‘PCR’. The smMIP method and its application for targeted transcriptome sequencing has been described before^20,21^ and is depicted in Fig. 1a. SmMIPs were designed against target regions of interest (UCSC human genome assembly hg19, and variant-specific FASTA sequences) with ligation and extension probes localized on different exons, depending on the splice variant to be detected, and leaving a gap of maximum 112 nt. SmMIPs were designed based on the MIPgen algorithm as described by Boyle *et al*.^33^ and added to a large panel of previously designed smMIPs^21^. The panel of phosphorylated smMIPs was hybridized to cDNA after which the gap was filled by primer extension and ligation. Non-reacted smMIPs and remaining RNA and cDNA were removed by exonuclease treatment, followed by PCR amplification of the circularized smMIP library using a unique barcoded reverse primer for each sample. After library pooling and purification using AMPureXP beads (Beckman Coulter Genomics, High Wycombe, UK) smMIP-PCR libraries were sequenced on the Illumina Nextseq platform (Illumina, San Diego, CA) at the Radboudumc sequencing facility. Reads were mapped against the reference transcripts (UCSC human genome assembly hg19 and variant-specific FASTA sequences) using the SeqNext module of JSI SequencePilot version 4.2.2 build 502 (JSI Medical Systems, Ettenheim, Germany). The random 8xN nucleotide tag flanking the ligation probe was used to reduce PCR amplicates to one consensus read originating from the same smMIP (unique read). Read counts for each smMIP were normalized to the total read count within a sample and multiplied by 10^6^ (Fragments per Million, FPM). Individual transcript levels were expressed as mean FPM of all smMIPs targeting that transcript.

SmMIPs were designed against transcript variants of MET (total MET, MET^Δ7-8^, MET^Δ14^) and EGFR (total EGFR, EGFRvIII). SmMIP sequences are depicted in Table 1. An example of smMIP design for total MET (transcript variant 2) and MET^Δ7-8^ is depicted in Fig. 1b. To determine the cumulated expression of all MET transcript variants, smMIPs were designed against shared parts of the transcripts (total MET). To enable detection of truncated transcripts, smMIPs were designed against splice variant-specific exon-exon junctions, either with the maximum 112 nt gap or the ligation/extension probe covering the exon-exon junction (exon 6–9 junction for MET^Δ7-8)^. SmMIPs were designed in a similar way for MET^Δ14^, total EGFR and EGFRvIII.

**Table 1:** SmMIP nucleotide sequences. Ligation and extension probes are depicted in Italic.

Transcript	Exon targets		MIP sequence (5′→3′)
	Extension	Ligation	
variant	probe	probe	
Total MET	Exon 9	Exon 9–10	*GTGGTGGGAGCACAATAACAGGTGTTG*NNNNNNNNCTTCAGCTTCCCGATATCCGACGGTAGTGT*CAAACCATTTCAACTGAG*
(variant 2)	Exon 9	Exon 11	*GCATGTCAACATCGCTCTAATTCAG*NNNNNNNNCTTCAGCTTCCCGATATCCGACGGTAGTGT*ATTCATCCAACCAAATCTTT*
MET^Δ7-8^	Exon 6	Exon 6–9	*GCACGATGAATACTGTGTCAAACAG*NNNNNNNNCTTCAGCTTCCCGATATCCGACGGTAGTGT*TTGAAGGAGGGACAAGGCTG*
	Exon 6	Exon 9	*GCCAACCGAGAGACAAGCATCTTCA*NNNNNNNNCTTCAGCTTCCCGATATCCGACGGTAGTGT*AATGAGAGCTGCACCTTGAC*
MET^Δ14^	Exon 13	Exon 15	*GTTTCCTAATTCATCTCAGAACGGTTCA*NNNNNNNNCTTCAGCTTCCCGATATCCGACGGTAGTGT*AATAGTTCAACCAGATC*
	Exon 13	Exon 15	*CAGTCCATTACTGCAAAATACTGTCCACA*NNNNNNNNCTTCAGCTTCCCGATATCCGACGGTAGTGT*AAAAAGAGAAAGCAAA*
Total EGFR	Exon 1	Exon 2	*GGAAATTACCTATGTGCAGAGGAATTATG*NNNNNNNNCTTCAGCTTCCCGATATCCGACGGTAGTGT*CTGGAGGAAAAGAAAG*
	Exon 3	Exon 3	*GCAAATAAAACCGGACTGAAGGAGC*NNNNNNNNCTTCAGCTTCCCGATATCCGACGGTAGTGTGT*GGCTGGTTATGTCCTCAT*
	Exon 3	Exon 4	*GCCCTGTGCAACGTGGAGAGCATC*NNNNNNNNCTTCAGCTTCCCGATATCCGACGGTAGTGT*CTACGAAAATTCCTATGCCTT*
	Exon 5–6	Exon 6–7	*GCCTGGTCTGCCGCAAATTCCGAGACGAA*NNNNNNNNCTTCAGCTTCCCGATATCCGACGGTAGTGT*CCAGAAACTGACCAAA*
	Exon 7	Exon 8	*GCCTGTGGGGCCGACAGCTATGAGATGGA*NNNNNNNNCTTCAGCTTCCCGATATCCGACGGTAGTGT*TCTACAACCCCACCAC*
	Exon 9	Exon 10–11	*CGTAAAGGAAATCACAGGGTTTTTGCTGA*NNNNNNNNCTTCAGCTTCCCGATATCCGACGGTAGTGT*AAACACTTCAAAAACT*
	Exon 15	Exon 17	*GCCCTGGGGATCGGCCTCTTCAT*NNNNNNNNCTTCAGCTTCCCGATATCCGACGGTAGTGT*ACCTGTGCCATCCAAACTGCAC*
	Exon 17–18	Exon 18–19	*GCACGGTGTATAAGGGACTCTGGAT*NNNNNNNNCTTCAGCTTCCCGATATCCGACGGTAGTGT*TGCTGCAGGAGAGGGAGCTT*
	Exon 18	Exon 19–20	*GCCAACAAGGAAATCCTCGATGAAGC*NNNNNNNNCTTCAGCTTCCCGATATCCGACGGTAGTGT*AAAAGATCAAAGTGCTGGG*
	Exon 19	Exon 20	*TCTGCCTCACCTCCACCGTGCA*NNNNNNNNCTTCAGCTTCCCGATATCCGACGGTAGTGT*AAGTTAAAATTCCCGTCGCTATC*
	Exon 20	Exon 20	*GCTCCCAGTACCTGCTCAACTGGTGTGT*NNNNNNNNCTTCAGCTTCCCGATATCCGACGGTAGTGT*TGGACAACCCCCACGT*
	Exon 21	Exon 21–22	*GCAGAAGGAGGCAAAGTGCCTATCAA*NNNNNNNNCTTCAGCTTCCCGATATCCGACGGTAGTGT*AGGACCGTCGCTTGGTGCA*
	Exon 21	Exon 23	*GAGTTGATGACCTTTGGATCCAAGCC*NNNNNNNNCTTCAGCTTCCCGATATCCGACGGTAGTGT*CGGAAGAGAAAGAATACCA*
	Exon 23–24	Exon 25	*GCCAAGTCCTACAGACTCCAAC*NNNNNNNNCTTCAGCTTCCCGATATCCGACGGTAGTGTCAT*GGTCAAGTGCTGGATGATAG*
	Exon 26	Exon 27	*GACAGCATAGACGACACCTTCCTCCCAG*NNNNNNNNCTTCAGCTTCCCGATATCCGACGGTAGTGT*AGTGCAACCAGCAACAA*
	Exon 28	Exon 28	*GCCACCAAATTAGCCTGGACAACCCTG*NNNNNNNNCTTCAGCTTCCCGATATCCGACGGTAGTGT*AGAGACCCACACTACCA*
EGFRvIII	Exon 1	Exon 8	*GTGGTGACAGATCACGGCTCGTG*NNNNNNNNCTTCAGCTTCCCGATATCCGACGGTAGTGT*GAGAGCCGGAGCGAGCTCTT*
	Exon 1	Exon 8–9	*GCCGCAAAGTGTGTAACGGAATAGGTA*NNNNNNNNCTTCAGCTTCCCGATATCCGACGGTAGTGT*CTGGAGGAAAAGAAAG*

### Padlock probe design

The protocol for padlock probe rolling circle amplification and the design of padlock probes with associated bridged nucleic acid (BNA) primers was adapted from Weibrecht *et al*.^26^ and is summarized in Fig. 2a,b. Wild-type transcripts were distinctively detected with padlock probes targeting ‘normal’ exon-exon junctions that are missing in the exon-deletion transcript variants. For specific detection of splice variants, padlock probes were designed to target the splice variant-specific MET exon-exon junctions 6–9 (for detection of MET^Δ7-8^) and 13–15 (for detection of MET^Δ14^) and EGFR exon-exon junction 1–8 (for detection of EGFRvIII, Fig. 2b). To allow dual detection of splice variants together with their associated wild-type transcripts, specific sequences were included in the backbone of the padlock probe allowing detection via hybridization of complementary fluorescent probes. Bridged Nucleic Acid (BNA) primer, padlock probe and detection probe nucleotide sequences, as well as the nomenclature of probes and transcript variants used in this paper, are outlined in Table 2. Before use, padlock probes (IDT, Leuven, Belgium) were 5′-phosphorylated using 0.2 U/µl T4 PNK in 1x PNK buffer A (ThermoFisher Scientific, Waltham, MA, USA) according to manufacturer’s instructions.

**Table 2:** BNA primer, padlock probe and detection probe nucleotide sequences.

Transcript variant	Exon targets (probe)	Nucleotide sequences (5′→3′)		
		BNA primer	Padlock probe	Detection probe
METexon7-8^wt^	Exon 7-8 (MET^7,8^)	C**A**G**C**CAT**A**G**G**A**C**C **G**TATTTCGGCGA	*GATCCTGTAATAACAAGTAT*GTGACATACTGACAA***AGTAGCCGTG*** ***ACTATCGACT***TGACCAGTTAGCAAA*ACAGTACATTCTCCTATGTG*	Cy5-AGTAGCCGTGACTATCGACT
MET^Δ7-8^	Exon 6-9 fusion (MET^6–9^)	G**G**G**C**T**G**G**G**G**T**A**T**A **A**CATTCAAGAAT	*TGTGTCAAACAGTATTCTTGGT*GACATACTGACA*CCTCAATGCACA* *TGTTTGGCTCC*TGACCAGTTAGCA*AGTGAGAGCACGATGAATAC*	TexRd-XN-CCTCAATGCACATGTTTGGCTCC
METexon14^wt^	Exon 14 (MET^14^)	C**A**C**T**T**C**G**G**G**C**A**C**T**T** ACAAGCCTATC	*CACTCCTCATTTGGATAGGC*GTGACATACTGACAA***AGTAGCCGTG*** ***ACTATCGACTT***GACCAGTTAGCAAA*CGCTACGATGCAAGAGTACA*	Cy5-AGTAGCCGTGACTATCGACT
MET^Δ14^	Exon 13-15 fusion (MET^13–15^)	T**C**G**G**C**A**T**G**A**A**C**C**G **T**TCTGAGATGAA	*ATCAGTTTCCTAATTCATCT*GTGACATACTGACA*CCTCAATGCACAT* *GTTTGGCTCC*TGACCAGTTAGCA*AAAGAGAAAGCAAATTAAAG*	TexRd-XN-CCTCAATGCACATGTTTGGCTCC
EGFR^wt^	Exon 2-3 (EGFR^2,3^)	**A**G**G**G**C**A**A**T**G**A**G**G**A** CATAACCAGCCA	*ACCATCCAGGAGGTGGCTGG*GTGACATACTGACAA***AGTAGCCGT*** ***GACTATCGACT***TGACCAGTTAGCAAA*ATGATCTTTCCTTCTTAAAG*	Cy5-AGTAGCCGTGACTATCGACT
EGFRvIII	Exon 1-8 fusion (EGFR^1–8^)	C**T**C**G**G**A**C**G**C**A**C**G**A**G**CCGTGATCTGT	*GTAATTATGTGGTGACAGAT*GTGACATATGAAA*CCTCAATGCTGCTGC* *TGTACTAC* TGACCAGTTAGCA*GGCTCTGGAGGAAAAGAAAG*	TexRd-XN-CCTCAATGCTGCTGCTGTACTAC

BNA primer Bridged Nucleic Acids are depicted in bold. Padlock probe target-specific sequences are depicted in italic. Detection probe complementary sequences in the padlock probe are coloured bold italic (wild-type) or italic underline (mutant).

### Padlock probe rolling circle amplification

The protocol for padlock probe rolling circle amplification is summarized in Fig. 2a. For padlock assays on cell lines, cells were seeded on 8-well Lab-Tek II chamber slides (ThermoFisher Scientific, Waltham, MA, USA) and fixated the following day in 3.7% freshly prepared formaldehyde in DEPC-treated PBS (DEPC-PBS) for 30 min at room temperature. Fixated cells were washed twice with DEPC-PBS and dehydrated through an ethanol series (70%, 85% and 99.5%). Secure-seals (Grace Bio-Labs inc., Bend, Oregon, USA) with a volume of 50 µl (Ø 9 mm, 0.8 mm deep) were mounted on the Lab-Tek slides and cells were rehydrated by addition of DEPC-PBS-Tween (DEPC-PBS with 0.05% Tween-20). For tissue analyses, formalin-fixed paraffin-embedded (FFPE) sections of E98 xenografts (E98-FM, generated from the E98 cell line) and E468 xenografts were deparaffinized and fixated in 3.7% paraformaldehyde (PFA) in diethylpyrocarbonate (DEPC)-treated PBS. Tissue sections were permeabilized with pepsin (2 mg/ml in 0.1 M HCl) and dehydrated through an ethanol series (70%, 85% and 99.5%). Secure-seals (Grace Bio-Labs inc., Bend, Oregon, USA) with a volume of 250 µl (Ø 20 mm, 0.8 mm deep) were mounted and cells were rehydrated with DEPC-PBS-Tween. All following incubation steps were performed within the secure seal chambers, incubated in a hybridizer (Dakocytomation, Glostrup, Denmark).

*In situ* reverse transcription was performed using BNA primers positioned within ≤1 nt from the 5′ end of the padlock probe target site (see Table 2 for nucleotide sequences). BNA primer hybridization and mRNA reverse transcription were performed o/n at 42 °C using 200 nM of BNA primer (IDT, Leuven Belgium), 20 U/µl RevertAid H minus M-MuLV reverse transcriptase in 1x M-MuLV reaction buffer (ThermoFisher Scientific, Waltham, MA, USA), 1 U/µl RiboLock RNase Inhibitor (ThermoFisher Scientific, Waltham, MA, USA), 500 µM dNTP and 0.2 µg/µl BSA (New England Biolabs, Ipswich, MA, USA). Post-fixation was performed with 3.7% formaldehyde (5 min) or 3.7% PFA (45 min) for cells or tissues, respectively. FFPE tissues were subsequently treated with glycine (2 g/100 ml in DEPC-PBS, 20 min RT) to decrease autofluorescence. Secure seal chambers were flushed with DEPC-PBS-Tween. Padlock probes (100 nM) were hybridized to the cDNA and ligated using 0.5 U/µl Ampligase in 1x Ampligase buffer (Epicentre, Madison, Wisconsin, USA) containing 0.4 U/µl RNase H (New England Biolabs, Ipswich, MA, USA), 1 U/µl RiboLock RNase Inhibitor, 0.2 µg/µl BSA, 50 mM KCl and 20% formamide (30 min 37 °C, 30 min 45 °C).

Rolling circle amplification of the ligated padlock probe was performed o/n at 37 °C using Phi29 DNA polymerase (ThermoFisher Scientific, Waltham, MA, USA). Secure seal chambers were flushed with DEPC-PBS-Tween and Phi29 DNA polymerase was added at a concentration of 1 U/µl in 1x Phi29 DNA polymerase buffer (ThermoFisher Scientific, Waltham, MA, USA) with 1 U/µl RiboLock RNase Inhibitor, 250 µM dNTP, 0.2 µg/µl BSA and 5% glycerol. After washing with DEPC-PBS-Tween, rolling circle products (RCPs) were visualized by hybridization to 100 nM of corresponding fluorescent detection probe (See Table 2) (IDT, Leuven, Belgium) in 1x saline-sodium citrate (SSC) and 20% formamide (37 °C, 30 min). The slides were dehydrated through an ethanol series (70%, 85% and 99.5%) and mounted with Vectashield (Vector Laboratories Inc., Burlingame, CA, USA) containing 375 ng/ml DAPI for nuclear counterstaining.

Slides were imaged using the Vectra (Caliper Life Sciences, Waltham, MA, USA). Slide analysis was performed with the Vectra Automated Quantitative Pathology Imaging System (Version 3.0.3, PerkinElmer Inc., Waltham, MA, USA) and inForm Advanced Image Analysis Software (Version 2.2.1, PerkinElmer Inc., Waltham, MA, USA). Contrast-enhancement was applied on the resulting images using GIMP version 2.8.20.

### Data availability

The datasets generated during and/or analysed during the current study are available from the corresponding author on reasonable request

## Electronic supplementary material

Supplementary data
