# Supplementary material for: Quantification and localization of oncogenic receptor tyrosine kinase variant transcripts using molecular inversion probes
Source: Sci Rep. 2018 May 4;8:7072. doi: 10.1038/s41598-018-25328-5 (PMC5935718; doi:10.1038/s41598-018-25328-5)

Quantification and localization of oncogenic receptor tyrosine kinase variant transcripts using molecular inversion probes

Corina N.A.M. van den Heuvel[1], Arvid I. Das[2], Tessa de Bitter[2], Femke Simmer[2], Thomas Wurdinger[3], Miguel Angel Molina-Vila[4], William P.J. Leenders[1]

[1] Department of Biochemistry, Radboud Institute for Molecular Life Sciences, University Medical Centre, Nijmegen, 6525 GA, The Netherlands and [2]Department of Pathology, Radboud University Medical Centre, Nijmegen, 6500 HB, The Netherlands, [3]VU Medical Centre Amsterdam, Department of Neurosurgery, Amsterdam, The Netherlands, [4]PangaeaOncology, Laboratory of Oncology, University Hospital Quiron Dexeus, Barcelona, Spain.

Supplementary Information file

Supplementary Figure 1 (supplementary data to Figure 3)

a

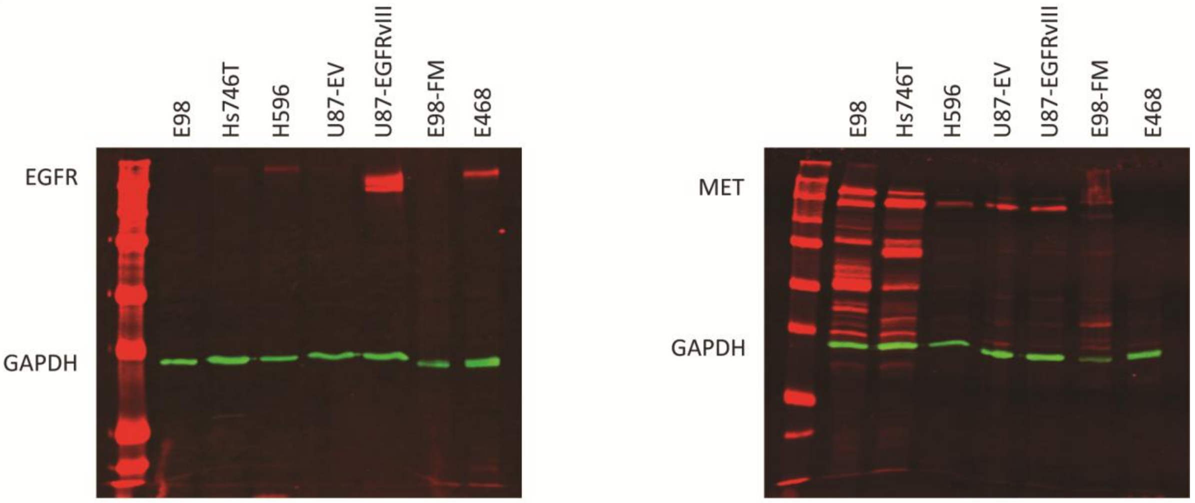

b

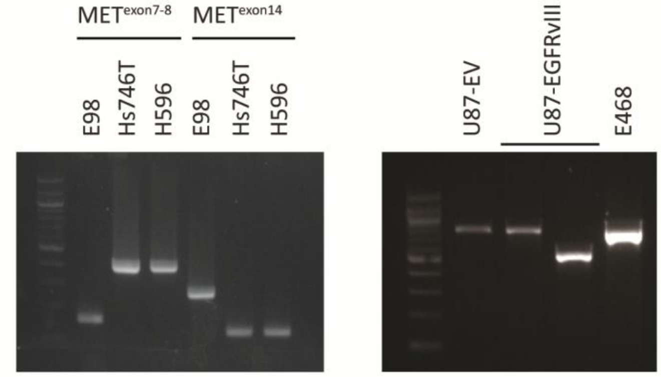

Supplementary Figure 2 (supplementary data to Figure 6d)

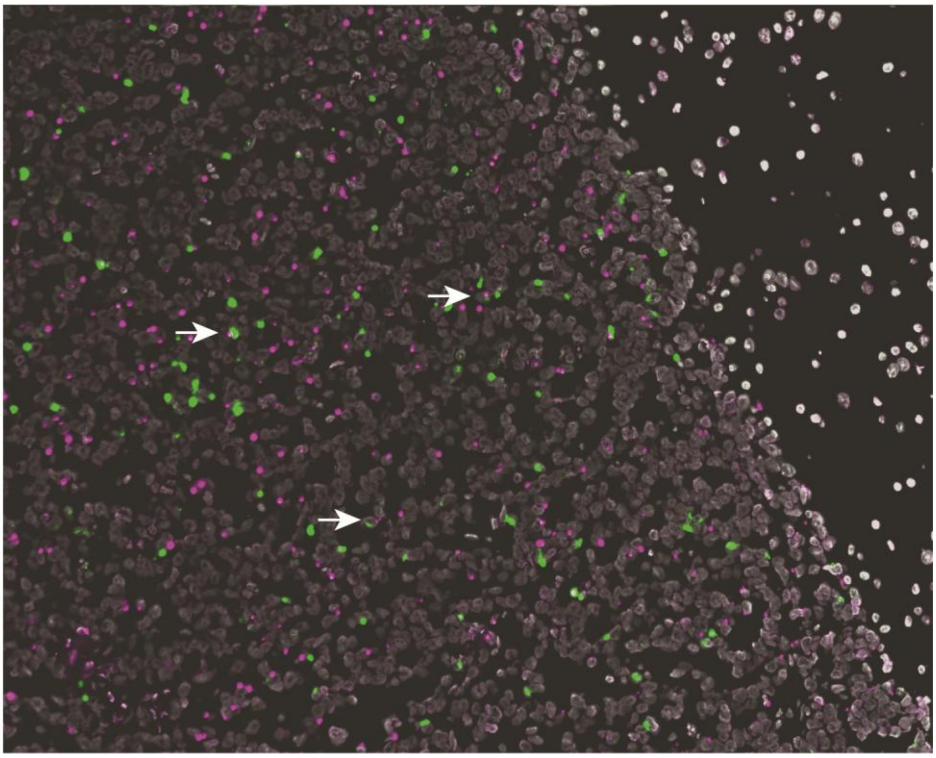

Supplement: Supplementary file 1 — Supplementary data [file 41598_2018_25328_MOESM1_ESM.pdf]
